# HMGB1 Recruits TET2/AID/TDG to Induce DNA Demethylation in STAT3 Promoter in CD4^+^ T Cells from aGVHD Patients

**DOI:** 10.1155/2020/7165230

**Published:** 2020-09-24

**Authors:** Xuejun Xu, Yan Chen, Enyi Liu, Bin Fu, Juan Hua, Xu Chen, Yajing Xu

**Affiliations:** Department of Hematology, Xiangya Hospital, Central South University, Changsha, Hunan, China

## Abstract

STAT3 is highly expressed in aGVHD CD4^+^ T cells and plays a critical role in inducing or worsening aGVHD. In our preceding studies, DNA hypomethylation in STAT3 promoter was shown to cause high expression of STAT3 in aGVHD CD4^+^ T cells, and the process could be modulated by HMGB1, but the underlying mechanism remains unclear. TET2, AID, and TDG are indispensable in DNA demethylation; meanwhile, TET2 and AID also serve extremely important roles in immune response. So, we speculated these enzymes involved in the STAT3 promoter hypomethylation induced by HMGB1 in aGVHD CD4^+^ T cells. In this study, we found that the binding levels of TET2/AID/TDG to STAT3 promoter were remarkably increased in CD4^+^T cells from aGVHD patients and were significantly negatively correlated with the STAT3 promoter methylation level. Simultaneously, we revealed that HMGB1 could recruit TET2, AID, and TDG to form a complex in the STAT3 promoter region. Interference with the expression of TET2/AID/TDG inhibited the overexpression of STAT3 caused by HMGB1 downregulation of the STAT3 promoter DNA methylation. These data demonstrated a new molecular mechanism of how HMGB1 promoted the expression of STAT3 in CD4^+^ T cells from aGVHD patients.

## 1. Introduction

Allogeneic hematopoietic stem cell transplantation (allo-HSCT) has been recognized as the exclusive treatment to cure hematopoietic malignancies, but acute graft-versus-host disease (aGVHD) is the primary limitation of the therapy [1–3]. Over the past decade, despite significant improvements in allo-HSCT, aGVHD remained the leading cause of transplant-related morbidity and mortality [4, 5].

Signal transducer and activator of transcription 3 (STAT3), an important signal transducer and activator of transcription, participates in regulating various biological processes [6]. Overexpressed STAT3 in aGVHD was found to be tightly linked to various disease progression [7, 8]. Our previous study showed the significantly increased expression of STAT3 was associated with DNA hypomethylation in STAT3 promoter in aGVHD CD4^+^ T cells.

HMGB1, a group of nonhistone nucleoproteins, involves in mediating transcription and inflammatory processes [9, 10]. HMGB1 was found to drive DNA demethylation in CD4^+^ T cells of systemic lupus erythematosus (SLE) patients [11]. DNA methylation is an epigenetic mechanism involved in regulating the gene expression [12]. The gene promoter hypermethylation could reduce gene expression, and conversely, demethylation of the promoter increased the gene expression [13, 14]. Our previous study revealed that HMGB1 was markedly overexpressed in CD4^+^ T cells from aGVHD patients and was positively correlated with the STAT3 promoter DNA methylation level [7]. However, the exact mechanism by which HMGB1 decreases the DNA methylation level of STAT3 promoter remains unclear.

There are two pathways of DNA demethylation: passive and active [15]. During the cell division, DNA methyltransferases (DNMTs) are suppressed, which gradually lead to the decrease of the DNA methylation level [16]. This process is defined as passive DNA demethylation. Ten-eleven translocation (TET), Activation-induced cytidine deaminase (AID), and thymine-DNA glycosylase (TDG) are essential for active DNA demethylation in mammalian cells [17, 18]. 5-Methylcytosine (5-mC) can be turned into 5-hydroxymethylcytosine (5-hmC), 5-formylcytosine (5-fC), and 5-carboxylcytosine (5-caC) by TET proteins. Then, 5-hmC is catalyzed by AID into 5-hydroxymethyluracil (5hmU). Next, 5hmU is subsequently reduced to cytosine (C) by base excision repair (BER), which is induce by TDG. Moreover, 5-fC and 5-caC can be directly reduced to C by TDG. Through these two modes of action, the three enzymes initiate and maintain active DNA demethylation. So, we speculated that these enzymes stand a good chance of involving in the process that HMGB1 induced demethylation of STAT3 promoter in CD4^+^ T cells from aGVHD patients.

In this study, we explored the specific process by which HMGB1 increases the expression of STAT3 in CD4^+^ T cells from aGVHD patients and finally confirmed that HMGB1 could extensively recruit TET2, AID, and TDG to bind to STAT3 promoter, which in turn contributed to DNA demethylation of STAT3 promoter. Taken together, the result of this study uncovered the novel molecular mechanism of STAT3 demethylation induced by HMGB1 in aGVHD CD4^+^ T cells.

## 2. Materials and Methods

### 2.1. Patients

A total of 46 patients who underwent allo-HSCT between 2017 and 2019, from HLA-identical sibling donors at the Central of Hematopoietic Stem Cell Transplantation of Xiangya Hospital, were included in this study. This study was approved by the human ethics committee of the Xiangya Hospital of Central South University, and written informed consent was obtained from all subjects. The clinical characteristics of these patients are shown in Table 1. The median time from transplantation onset to the start of aGVHD was 50 (21-87) days. The conditioning regimes were adopted as described in our previous study [7]. Assessment of aGVHD was conducted based on clinical symptoms in accordance with the accepted criteria [19]. The patients were divided into two groups according to whether or not they suffered from aGVHD. We simultaneously collected samples from patients at the onset of aGVHD (*n* = 23) and patients without aGVHD (*n* = 23). When patients were diagnosed with aGVHD, the blood samples were collected before treatment.

### 2.2. Culturing and Transfection of Cells

CD4^+^ T cells were extracted from 40 ml venous peripheral blood using human CD4 beads (Miltenyi, Bergisch Gladbach, Germany) and cultured in human T cell culture medium (Lonza, Walkersville, MD, USA) supplemented with 10% fetal bovine serum (FBS) and 1% penicillin-streptomycin. CD4^+^ T cells were transfected using the human T cell nucleofector kit and Amaxa nucleofector (Lonza, Walkersville, MD, USA). Briefly, CD4^+^ T cells were collected and resuspended in 100 *μ*l human T cell nucleofector solution, and then the cell suspension was mixed with plasmids. The mix was electrotransfected using the nucleofector program V-024 in the Amaxa nucleofector. The transfected cells were cultured in human T cell culture medium and harvested after 48 h. Jurkat cells were cultured in the RPMI 1640 media (Gibco, Rockville, MD, USA) containing 10% FBS and incubated at 37°C in 5% CO_2_. The plasmids were transfected into Jurkat cells via electrotransfection as described above.

### 2.3. Western Blotting

The detailed procedure western blotting was performed as previously reported [7]. CD4^+^ T cells were lysed in 1% NP40 lysis buffer [20 mM Tris/HCl (pH 7.2), 200 mM NaCl, 1% NP40] containing proteinase inhibitor (Thermo Pierce). Lysates were centrifuged at 12,000 g for 15 min at 4°C, and protein concentration was detected by the Bradford protein assay (Bio-Rad, CA, USA). Equal amounts of proteins were separated on SDS-PAGE gels and then transferred to PVDF membranes (Bio-Rad, CA, USA). Membranes were blocked with 5% nonfat milk in Tris-buffered saline containing 0.1% Tween-20 (TBST) buffer and immunoblotted with primary antibodies, including anti-HMGB1 (Abcam, MA, USA), anti-TDG (Abcam, MA, USA), anti-AID (Cell Signaling, BSN, USA), anti-TET2 (Abcam, MA, USA), anti-STAT3 (Cell Signaling, BSN, USA), and anti-GAPDH (Santa Cruz, CA, USA). Band intensity was quantified using Quantity One software (Bio-Rad, CA, USA).

### 2.4. ChIP-Real Time PCR

CD4^+^ T cells were incubated in media with 1% formaldehyde for 20 min at room temperature, and then crosslinking was stopped with glycine (final concentration, 0.125 M) for 5 min. Cells were collected after washing twice, suspended in cold RIPA buffer [10 mM Tris-Cl (pH 8.0), 150 mM NaCl, 0.1% SDS, 0.1% DOC, 1% Triton X-100, 5 mM EDTA], and sonicated to shear the genomic DNA. Next, anti-TDG, anti-AID, and anti-TET2 antibodies were added, and then the mixture was incubated overnight at 4°C. Protein A agarose beads were added to collect the protein-DNA complexes. Samples were then washed and decrosslinked overnight at 65°C using sodium chloride (final concentration, 0.2 M). Finally, enriched DNA was recovered and amplified by real-time PCR. The ChIP-qPCR primers for STAT3 promoter are presented in Table 2.

### 2.5. Coimmunoprecipitation (co-IP)

Cell proteins were extracted by RIPA lysis buffer. Then, anti-HMGB1 antibody was added and incubated overnight at 4°C. Protein A/G agarose beads were added to samples and incubated for 2 hours at room temperature. Agarose beads were harvested by centrifugation at 3000 g for 2 min. Finally, the protein complex was eluted with loading buffer and analyzed by western blotting. Primary antibodies included anti-TET2, anti-AID, and anti-TDG.

### 2.6. Bisulfite Sequencing

Genomic DNA was extracted from CD4^+^ T cells using the TIANamp genomic DNA kit (TIANGEN, Beijing, China). The EpiTect bisulfite kit (Qiagen, CA, USA) was utilized to convert bisulfite. Three CpG islands within the STAT3 promoter region were amplified by PCR. The PCR products were subcloned into a pGEM-T vector (Promega, WI, USA). Ten independent clones were sequenced for each amplified fragments. Primers used were as follows:

5′-GAATATTTTATGTATTTTA-3′ (forward 1) and 5′-ACAACAAAAAAAACATA-3′ (reverse 1); 5′-AGTTGTTTTTTTTATTGGT-3′ (forward 2) and 5′-CCCTACACCCCCTTCACC-3′ (reverse 2); 5′-GGGATTTTGGGGATGTTG-3′ (forward 3) and 5′-AAAAAACACAACTATCT-3′ (reverse 3).

### 2.7. Statistical Analysis

Variables were analyzed by Student's *t*-test (two groups) or single-factor analysis of variance (three groups). Correlations were analyzed using Pearson's correlation coefficient. All analyses were performed with SPSS 22.0 software. Statistical significance was set at *p* < 0.05.

## 3. Results and Discussion

### 3.1. HMGB1, TET2, AID, and TDG Bind to STAT3 Promoter

**Figure 1:**
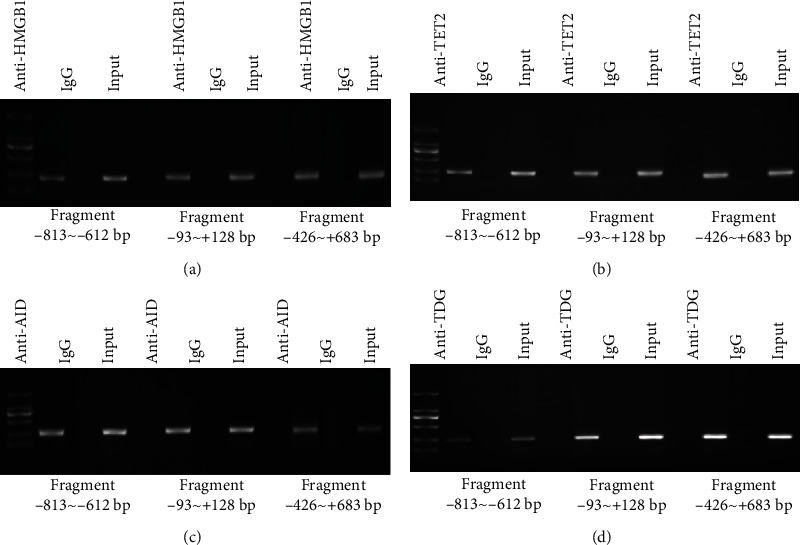
HMGB1, TET2, AID, and TDG were detected in STAT3 promoter. ChIP-PCR showed that HMGB1, (a) TET2, (b) AID, and (c) TDG bound to the STAT3 promoter region (-813 bp to +683 bp).

Our previous study showed that HMGB1 could induce DNA demethylation of STAT3 promoter to worsen aGVHD [7]. Hence, we hypothesized that some methylation-related enzymes played crucial roles in this process. We first explored whether HMGB1, TET2, AID, and TDG could bind to STAT3 promoter using a ChIP-PCR analysis in HMGB1/TET2/AID/TDG overexpressed Jurkat cells. Three pairs of primers that covered the STAT3 promoter −813 bp to +683 bp region were used. The results revealed that HMGB1, TET2, AID, and TDG could indeed bind to the STAT3 promoter −813 bp to +683 bp region (Figures 1(a)–1(c)).

### 3.2. Binding Levels of TET2, AID, and TDG in STAT3 Promoter Were Obviously Enhanced in CD4^+^ T Cells from aGVHD Patients

**Figure 2:**
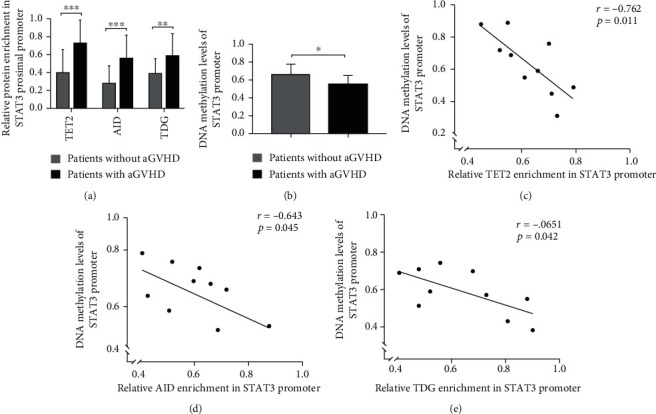
Binding level of TET2/AID/TDG in STAT3 promoter was extremely upregulated in aGVHD CD4^+^ T cells. (a) ChIP-qPCR analysis of the binding level of TET2/AID/TDG in the STAT3 promoter region in CD4^+^ T cells from patients with (*n* = 23) or without (*n* = 23) aGVHD. (b) DNA methylation level of STAT3 promoter in CD4^+^ T cells from aGVHD patients (*n* = 10) or non-aGVHD patients (*n* = 10) (0 = unmethylated; 0.5 = 50%methylated). (c–e) Correlation between relative TET2, AID, and TDG enrichment and DNA methylation in STAT3 promoter in aGVHD-CD4^+^ T cells (*r* = −0.762, *p* = 0.011; *r* = −0.643, *p* = 0.045; *r* = −0.651, *p* = 0.042; *n* = 10) (0 = unmethylated; 0.5 = 50%methylated); ^∗^*p* < 0.05, ^∗∗^*p* < 0.01, ^∗∗∗^*p* < 0.001.

**Figure 3:**
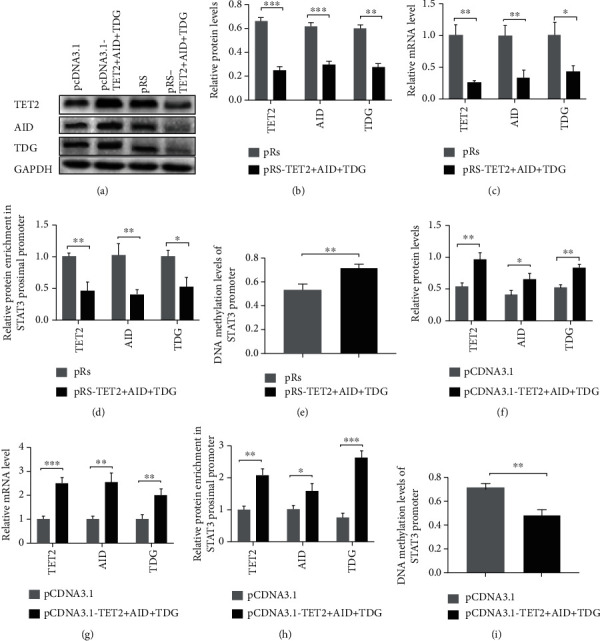
Interference with TET2/AID/TDG in aGVHD CD4^+^ T cells could induce DNA hypermethylation of STAT3 promoter. (a) Representative Western blot results for TET2, AID, TDG, and GAPDH levels. (b), (c) Quantitative analysis of the (b) relative protein levels and (c) relative mRNA levels of TET2, AID, and TDG in aGVHD CD4^+^ T cells transfected with TET2/AID/TDG interference plasmids or control plasmid. (d), (e) The binding levels of (d) TET2/AID/TDG in STAT3 promoter and (e) DNA methylation level of STAT3 promoter in TET2/AID/TDG-deficient aGVHD CD4^+^ T cells or control aGVHD CD4^+^ T cells. (f), (g) Quantitative analysis of the (f) relative protein levels and (g) relative mRNA levels of TET2, AID, and TDG in non-aGVHD CD4^+^ T cells transfected with TET2/AID/TDG overexpression plasmids or control plasmids. (h), (i) (h) The binding levels and (i) DNA methylation level in TET2/AID/TDG overexpressed non-aGVHD CD4^+^ T cells or control cells. Data represent the mean of three independent experiments (0 = unmethylated; 0.5 = 50%methylated); ^∗^*p* < 0.05, ^∗∗^*p* < 0.01, ^∗∗∗^*p* < 0.001.

We investigated whether the binding levels of TET2/AID/TDG in STAT3 promoter were significantly upregulated in CD4^+^ T cells of aGVHD patients. The binding levels of these proteins were measured by ChIP-qPCR in CD4^+^ T cells from patients with or without aGVHD. Compared with patients without aGVHD, the binding levels were remarkably increased in CD4^+^ T cells from aGVHD patients (Figure 2(a)). In addition, the DNA methylation of STAT3 promoter was detected in both groups.  Figure 2(b) shows marked hypomethylation in STAT3 promoter in CD4^+^ T cells from aGVHD patients as compared to non-GVHD CD4^+^ T cells. As shown in Figures 2(c)–2(e), the relative TET2/AID/TDG enrichment in STAT3 promoter was inversely correlated with DNA methylation level of STAT3 promoter in aGVHD CD4^+^ T cells. The core content of our study was to explore whether HMGB1 induced demethylation of STAT3 promoter by recruiting these DNA demethylases, and the three enzymes indeed participate in the process of DNA demethylation, and their functions are clear. In the other hand, certain studies have shown that TET, AID, and TDG could form complex to mediate the catalytic conversion of 5-methylcytosine to cytosine in the program of DNA demethylation. Therefore, the three proteins were interfered or overexpressed simultaneously in our experiment. Firstly, interference plasmids were transfected into aGVHD CD4^+^ T cells to repress the expression of TET2/AID/TDG (Figures 3(a)–3(c)). The binding levels of TET2/AID/TDG in STAT3 promoter were decreased after the TET2/AID/TDG interference in aGVHD CD4^+^ T cells (Figure 3(d)). Moreover, the DNA methylation level of STAT3 promoter was increased in aGVHD CD4^+^ T cells transfected with the TET2/AID/TDG interfering plasmid (Figure 3(e)). Next, the TET2/AID/TDG overexpression plasmids were transfected into non-aGVHD CD4^+^ T cells (Figures 3(f)–3(g)). The binding levels were upregulated after the overexpression of TET2, AID, and TDG in CD4^+^ T cells from non-aGVHD patients (Figure 3(h)). Simultaneously, the DNA methylation level of STAT3 promoter was significantly downregulated in non-GVHD CD4^+^ T cells with the overexpression of TET2/AID/TDG (Figure 3(i)). Taken together, excessive accumulation of TET2/AID/TDG complexes was observed in the STAT3 promoter region in aGVHD CD4^+^ T cells, which were strongly associated with DNA demethylation of STAT3 promoter.

### 3.3. HMGB1 Promoted TET2, AID, and TDG Binding to STAT3 Promoter

**Figure 4:**
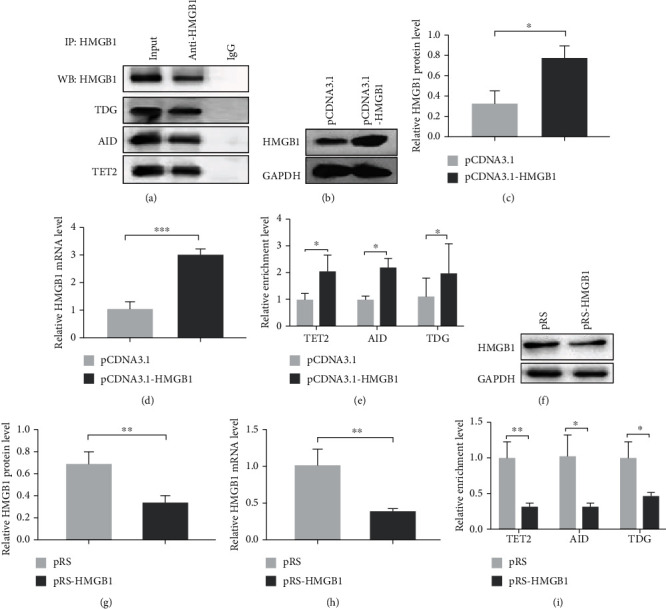
HMGB1 recruited TET2, AID, and TDG to bind to STAT3 promoter. (a) The combination of HMGB1 and TET2/AID/TDG was detected by Co-IP in Jurkat cells and analyzed by western blot analysis. (b–d) Representative western blotting results for (b) HMGB1, (c) relative HMGB1protein level, and (d) mRNA level in non-GVHD CD4^+^ T cells transfected with the HMGB1 overexpression plasmid or negative control plasmid. (e) The binding levels of TET2/AID/TDG in STAT3 promoter in HMGB1-overexpressed non-aGVHD CD4^+^ T cells and control cells. (f–i) After interference with HMGB1 in aGVHD CD4^+^ T cells, representative western blotting results for (f) HMGB1, (g) relative HMGB1 protein level, (h) relative HMGB1 mRNA level, and (i) the binding levels were detected. Experiments were repeated three times. Data are presented as the mean ± SD of three independent experiments. ^∗^*p* < 0.05, ^∗∗^*p* < 0.01.

HMGB1 increased the expression of STAT3 by modulating DNA demethylation of STAT3 promoter in CD4^+^ T cells, Hence, we investigated whether this process was correlated with TET2, AID, and TDG. Co-IP was performed in Jurkat cells to test whether HMGB1 form a complex with TET2, AID, and TDG. The results showed that HMGB1 coprecipitated with TET2, AID, and TDG (Figure 4(a)). Combine with Figure 1, we speculated that HMGB1 firstly bound to STAT3 promoter and then recruited TET2, AID, and TDG to form a complex in the promoter region to induce DNA demethylation. Next, we collected peripheral blood from patients with aGVHD or without aGVHD. pCDNA 3.1-HMGB1 was transfected into non-aGVHD CD4^+^ T cells to increase the HMGB1 expression (Figures 4(b)–4(d)). In comparison to the negative control group, the binding levels of TET2/AID/TDG in STAT3 promoter were significantly increased in HMGB1-overexpressed non-aGVHD CD4^+^ T cells (Figure 4(e)). Thereafter, HMGB1 was performed silenced in aGVHD CD4^+^ T cells (Figures 4(f)–4(h)). Compared with control cells, the binding levels reduced greatly in HMGB1-deficient aGVHD CD4^+^ T cells (Figure 4(i)). These data strongly demonstrated that HMGB1 could recruit TET2, AID, and TDG to bind to STAT3 promoter.

### 3.4. HMGB1 Regulated TET2/AID/TDG to Increase the STAT3 Expression through DNA Demethylation in STAT3 Promoter

**Figure 5:**
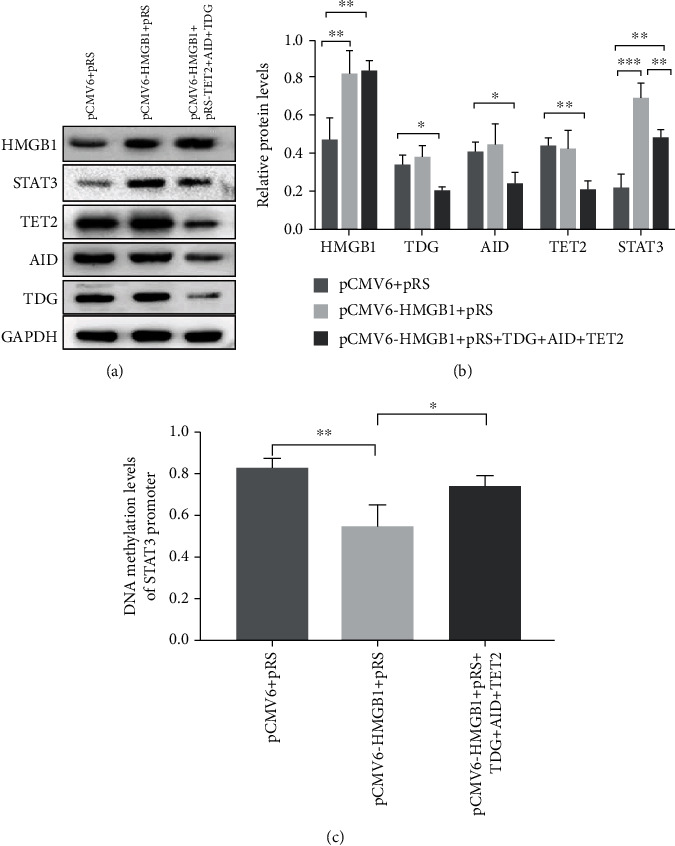
The overexpression of STAT3 induced by HMGB1 could be partially disrupted in CD4^+^ T cells transfected with the TET2/AID/TDG interference plasmid. (a) Representative western blot results for HMGB1, TDG, AID, TET2, STAT3, and GAPDH levels. (b) Quantitative analysis of the relative protein levels of HMGB1, TDG, AID, TET2, and STAT3. (c) DNA methylation levels of STAT3 promoter in CD4^+^ T cells transfected with different plasmids (control plasmids; HMGB1 overexpression plasmids; HMGB overexpression plasmids and TET2/AID/TDG interference plasmids); (0 = unmethylated, 1 = methylated). Data are presented as the mean ± SD of three independent experiments. ^∗^*p* < 0.05, ^∗∗^*p* < 0.01, ^∗∗∗^*p* < 0.001.

We hypothesized that TET2/AID/TDG played important roles in increasing the STAT3 expression, and the process was regulated by HMGB1 in aGVHD. Normal CD4^+^ T cells were divided into three groups based on different plasmid transfections (negative control plasmid; HMGB1 overexpression plasmid; HMGB1 overexpression plasmid and TET2/AID/TDG interference plasmid). The expression of the STAT3 and DNA methylation level of STAT3 promoter in CD4^+^ T cells from diverse groups was measured by Western blot and bisulfite sequencing (Figures 5(a)–5(c)). Compared with the negative control group, the STAT3 expression was significantly increased in CD4^+^ T cells transfected with HMGB1 overexpression plasmids. Strikingly, when the TET2/AID/TDG interference was superposed, the expression of STAT3 was also higher than that in the control group, while the expression sharply declined compared with the HMGB1-overexpressed group of CD4^+^ T cells. Meanwhile, the DNA methylation level of STAT3 promoter was majorly decreased after the overexpression of HMGB1 in normal CD4^+^ T cells. However, after the overexpression of HMGB1 and inhibition of TET2/AID/TDG in normal CD4^+^ T cells, the methylation level was not obviously different from the control group. Compared with HMGB1-overexpressed CD4^+^ T cells, the methylation level was increased in cells cotransfected with HMGB1 overexpression plasmids and TET2/AID/TDG interference plasmids. Taken together, these results demonstrated that HMGB1 could induce DNA demethylation of STAT3 promoter by recruiting TET2/AID/TDG. There may be other downstream molecules involved in the process by which HMGB1 facilitates the overexpression of STAT3 in aGVHD CD4^+^ T cells.

## 4. Discussion

DNA methylation is a powerful epigenetic mechanism, and its function seems to vary based on the surrounding environment [20]. The regulation of gene transcription or chromatin structure induced by DNA methylation participates in various pathological processes, such as inflammation, and human diseases including immunological diseases [21, 22]. For example, DNA hypomethylation in STAT3 promoters contributed to rheumatoid arthritis by controlling the activation and differentiation of immune cells [23]. Therefore, it was important to explore the function of DNA methylation in aGVHD.

Demethylases as the vital driving forces involve in the process of active DNA demethylation [16]. The discovery of the TET family proteins marked the beginning of a completely new chapter to the history of DNA demethylation, and the TET proteins are the key molecules to start the program of DNA demethylation [24]. This family include three members: TET1, TET2, and TET3; thereinto, TET2 is mainly expressed in the hemopoietic system [24, 25]. Mediating the catalytic conversion of 5-mC to 5-hmC, 5-fC ,and 5-caC is the prominent function of TET proteins in the process of active DNA demethylation [26]. AID participates in adaptive immune response, which is predominantly found in mature B cells [27]. AID regulates the hematopoietic system by influencing the differentiation of bone marrow cells and red cells [28]. Besides, TET2 and AID could form a complex to induce DNA demethylation in biological processes [28]. TDG, the pivotal enzyme of BER, act as an important downstream molecule of TET and AID to regulate demethylation of CpG sites in DNA [29, 30]. From the above, TET and AID play important roles in the hemopoietic system and immune system, and they also engage in DNA demethylation with TDG.

In addition, the enzymes are also relevant in the pathological processes. Increased 5-hmC, an important cause of overreactivity of CD4^+^ T cells, was correlated with upregulated TET2 in SLE patients [31]. Besides, the overexpression of TET2 promoted follicular helper-like T cells to worsen SLE via increasing some regulatory factors (sialophorin, signal transducing activator of transcription 5b and B cell lymphoma 6), and the process was associated with DNA demethylation of these factors [32, 33]. The functions of AID in autoimmune diseases are well established. AID heterozygous MRL/lpr mice survived longer as compared to MRL/lpr mice, a significant model of SLE [34]. The excessive autoantibodies were correlated with the overexpression of AID in BXD2 mice, which are susceptible to autoimmune diseases [35]. Furthermore, the TET2/AID complex, which bound to the FA complementation group A (FANCA) promoter and induced its hypomethylation, facilitated oncogenic FANCA in diffuse large B cell lymphoma [36]. Our present study confirmed that TET2, AID, and TDG could form a complex, which was involved in DNA demethylation of STAT3 promoter in aGVHD-CD4^+^ T cells.

HMGB1, derived from HMGB family, is ubiquitously expressed in whole adult tissues and plays a critical role in gene transcription [37, 38]. HMGB1 assists other molecules such as organic cation/carnitine transporter1/2 and p53 to bind to target genes and facilitates DNA modification [39, 40]. HMGB1 could boost the Matn1 promoter activity via SRY-related high-mobility group box (SOX) trio in early chondrogenesis, and the binding efficiency of SOX trio in Matn1 promoter could be affected by HMGB1 [41]. Furthermore, HMGB1 formed complexes with proinflammatory factors to involve in inflammatory pathologies [42]. Reduction of HMGB1 could inhibit the expression of inflammatory cytokines [43]. Our results showed that HMGB1 increased the expression of STAT3 via recruiting TET2/AID/TDG to decrease DNA methylation level of STAT3 promoter. Moreover, there are other mechanisms underlying the overexpression of STAT3 induced by HMGB1 in CD4^+^ T cells from aGVHD patients.

Moreover, the DNA methylation level continually undergoes a dynamic change. Cytosine is converted to 5-methylcytosine by DNA methyltransferases (DNMTs) using S-adenosyl-L-methionine to offer methyl to the C5 position of cytosine [44]. DNMTs play critical roles in the physiological and pathological processes. Varun et al. have shown that the methylation level of conserved noncoding sequence 2 (CNS2) played a decisive role to the Foxp3 expression, which was the critical factor to differentiation and maintenance function of Treg cells [45, 46]. The research exposed that TET2 and DNMTs were competitive and could not simultaneously bind to CNS2 [45]. When TET2 combined with CNS2, DNMTs could not make contact with CNS2, and the expression of Foxp3 or Treg cells will be at an advantage [45]. With the growth of the Treg cells, the disease severity will be gradually decreased in aGVHD patients. In addition, 5-Azac (5-azacytidine), the inhibitor of DNMTs, was utilized to prevent aGVHD in clinic and achieved good performance [47]. Inducing the demethylation of Foxp3 was an important function of 5-Azac in aGVHD [48]. From above studies, DNMTs will be inhibited in aGVHD patients with 5-Azac, and it may promote the combination between TET2 and CNS2. All the changes will increase Tregs to prevent aGVHD. On the contrary, our research revealed that massive TET2 aggravate progress of aGVHD by decreasing DNA methylation of STAT3 promoter. It is hint that the specific effect of demethylase or DNMTs in the disease process are dependent on their target gene. And to study the molecules, the upstream or downstream regulatory factors of the enzymes are of great practical significance. With the advent of the era of precision medicine, we have realized that more and more patients benefit from molecularly targeted drugs and personalized gene therapies. Therefore, exploring the precise effects of molecules in the pathological process may provide a new therapeutic target for disease prevention and treatment.

## 5. Conclusion

This study identified a mechanism of STAT3 over-expression in CD4^+^ T cells from aGVHD patients. HMGB1 could recruit TET2, AID and TDG to facilitate expression of STAT3 via DNA hypomethylation of STAT3 promoter in aGVHD CD4^+^ T cells. Suppression of TET2/AID/TDG expression through interference plasmids could partially decrease over-expression of STAT3 induced by HMGB1. These findings provide a theoretical basis to investigate new therapeutic targets for aGVHD prevention and treatment.

## Figures and Tables

**Table 1:** Clinic characteristics of patients.

	Total	Male	Median age	Diagnosis				Days to aGVHD onset median (range)
				ALL	AML	CML	MDS	
Non-aGVHD	23	13	33	7	10	4	2	
aGVHD	23	12	32	7	12	3	1	50 (21-87)

ALL: acute lymphoblastic leukemia; AML: acute myeloid leukemia; CML: chronic myeloid leukemia; MDS: myelodysplastic syndrome.

**Table 2:** ChIP-qPCR primers for STAT3 promoter.

Segment position	Forward	Reverse	Product length
+71~+318	5′AGGAGCACCGAACTGTC-3′	5′-GCCCACTGACCAATGAG-3′	247
–226~–125	5′-GAGGGAACAAGCCCCAA-3′	5′-ACATCCCCAAGGTCCCA-3′	101
–2039–1754	5′-GGGTTGTGGAGAAAGGC-3′	5′-CATATTATCCGCTGATAG-3′	285

## Data Availability

The data to support the findings of this study are available from the corresponding author upon request.
